# Effects of Roasting on the Antioxidant and Lipid‐Modulating Activities of *Argania spinosa* Oil Under Diabetic Conditions

**DOI:** 10.1002/fsn3.72441

**Published:** 2026-10-06

**Authors:** Nour Elhouda Daoudi, Mohammed Aziz, Mohammed Choukri, Youssouf Ali Younous, Amir Bouallegue, Gehan M. Elossaily, Abdel‐Rhman Z. Gaafar, Musa A. Said, Hina Ali, Mohamed Bnouham

**Affiliations:** ^1^ Higher Institute of Nursing Professions and Health Techniques Oujda Morocco; ^2^ Laboratory of Bioresources, Biotechnology, Ethnopharmacology and Health, Department of Biology, Faculty of Sciences Mohammed First University Oujda Morocco; ^3^ Biochemistry Laboratory Central Laboratory Service—Centre Hospitalier Universitaire, Mohammed VI Oujda Morocco; ^4^ Evangelical College N'Djamena Chad; ^5^ Laboratory of Food Oral Processing, School of Food Science and Biotechnology Zhejiang Gongshang University Hangzhou China; ^6^ Department of Basic Medical Sciences, College of Medicine AlMaarefa University Riyadh Saudi Arabia; ^7^ Research Center, Deanship of Scientific Research and Post‐Graduate Studies AlMaarefa University Riyadh Saudi Arabia; ^8^ Department of Botany and Microbiology, College of Science King Saud University Riyadh Saudi Arabia; ^9^ Department of Chemistry, Faculty of Science Islamic University of Madinah Madinah Saudi Arabia; ^10^ Shanghai Key Laboratory for Molecular Engineering of Chiral Drugs, School of Chemistry and Chemical Engineering Shanghai Jiao Tong University Shanghai People's Republic of China

**Keywords:** *Argania spinosa* L., diabetes, diabetic complications, roasting process, vegetable oils

## Abstract

*Argania spinosa* L. is endemic to a small region in southwestern Morocco and has been used for food and medicinal purposes for decades. This study evaluates the hepatoprotective and potential cardioprotective effects of roasted Argan seed oil (HGAT) and non‐roasted Argan seed oil (HGANT) in a streptozotocin‐induced experimental diabetic model. An experimental approach in diabetic rats treated with HGAT or HGANT was used to assess oxidative stress, liver enzymes, and lipid metabolism. Diabetic rats were administered 2 mL/kg body weight/day of HGAT or HGANT. Biochemical analyses measured blood glucose levels, liver enzyme activity (ALT, AST, LDH), oxidative stress markers (MDA, GSH, CAT), lipid profiles, and hepatic glycogen content. Histopathological examinations assessed collagen deposition and fibrosis in liver and heart tissues. Both HGAT and HGANT significantly reduce oxidative stress and improve lipid profiles in diabetic rats. Notably, 2 mL/kg body weight/day of HGAT treatments decreased blood sugar levels to values comparable to those observed with metformin (*p* > 0.05). Histopathological analysis revealed that both oils improved hepatic architecture, reduced lipid droplet accumulation, and decreased collagen deposition in the liver. In cardiac tissue, HGAT and HGANT improved myocardial structure and significantly reduced collagen deposition, indicating attenuation of cardiac fibrosis. The roasting process did not affect total cholesterol, triglycerides, hepatic glycogen, or oxidative stress markers. Argan oil demonstrates promising hepatoprotective and lipid‐modulating effects in diabetic conditions, with additional cardioprotective potential, supporting its ethnomedicinal use in metabolic disorders. Further studies are warranted to clarify its underlying mechanisms, long‐term effects, and potential interactions with standard antidiabetic therapies.

## Graphical abstract

Roasted and non‐roasted *Argania spinosa* seed oils were evaluated in streptozotocin‐induced diabetic rats. Biochemical and histopathological analyses assessed glucose regulation, lipid metabolism, oxidative stress, liver enzymes, glycogen content, and tissue architecture. Both oils improved metabolic and hepatic parameters and attenuated cardiac fibrosis, supporting their preventive potential in diabetes‐related complications.

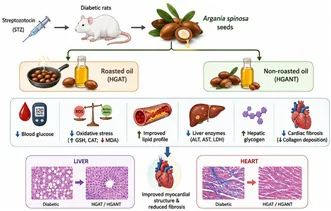

**Abbreviations**
ALT: alanine aminotransferase
AST: aspartate aminotransferase
CAT: catalase
DTNB: 5,5‐dithio‐bis‐(2‐nitrobenzoic acid)
GSH: reduced glutathione
H_2_O_2_
: hydrogen peroxide
HDL‐c: high density lipoproteins
HE: hematoxylin–eosin
HGANT: unroasted Argan seed oil
HGAT: roasted Argan seed oil
KOH: potassium hydroxide
LDH: lactate dehydrogenase
LDL‐c: plasma low density lipoproteins
MDA: malondialdehyde
STZ: streptozotocine
TBA: thiobarbituric acid
V_LDL‐c_
: very low density lipoproteins

## 1 Introduction

Diabetes is a chronic metabolic disorder characterized by persistent hyperglycemia due to abnormalities in insulin secretion, insulin action, or both. In Type 1 diabetes, hyperglycemia mainly results from the loss of pancreatic β‐cells and the consequent lack of insulin production, whereas Type 2 diabetes is primarily associated with insulin resistance together with a relative impairment of insulin secretion (Sabir et al. 2025). This insufficiency leads to an imbalance in the body's homeostasis, particularly affecting the vascular and nervous systems (Daoudi et al. 2022). Unfortunately, over the past few years, the incidence of diabetes has seen a remarkable increase, and it is projected that it will reach 784 million people by 2040 (International Diabetes Federation 2021). In this context, the use of medicinal plants as therapeutic alternatives is currently on the rise in the healthcare system, due to their richness in active principles, which could lead to the discovery of new medications to treat various diseases, including diabetes. Notably, diabetic cardiomyopathy, marked by ventricular hypertrophy, myocardial fibrosis, and impaired ventricular functions, along with an increased risk of liver diseases, represents significant health challenges for individuals with Type 2 diabetes (Tian et al. 2017).

Vegetable oils are plant‐derived lipids that provide essential energy and nutritional value. They serve as a significant source of fatty acids and vitamins, particularly vitamin D, which contributes to the regulation of phosphocalcic balance and the proper functioning of pancreatic β‐cells (American Diabetes Association 2002). As well as tocopherols that exhibit inhibitory activity against lipid peroxidation through free radical neutralization, thereby mitigating oxidative stress (Khallouki et al. 2003; Pazdro and Burgess 2010). The unsaturated fatty acids abundant in these oils are beneficial for preventing chronic diseases, particularly diabetes, and improving lipid and carbohydrate metabolism in both healthy and diabetic subjects (Wheeler et al. 2012). Many pharmacological studies have highlighted the effectiveness of vegetable oils in treating and preventing diabetes and its complications, with Argan oil being a notable example.

*Argania spinosa* L., an endemic species of Morocco, is renowned for the oils extracted from its fruit seeds. It is predominantly distributed in the regions of Souss, Essaouira, Tindouf, and Bni‐Znassen (Azizi et al. 2022; Hmamouchi 2001). For centuries, Argan has been used as a food source and is traditionally employed to treat various disorders such as diabetes, hypercholesterolemia, liver diseases, dry skin, juvenile acne, and skin blemishes. Pharmacological studies have shown that Argan oil has antioxidant (Drissi et al. 2004), nephroprotective (Daoudi, Mamri, et al. 2025), anti‐inflammatory, antihypertensive (Berrougui et al. 2004), anti‐obesity (Adlouni et al. 2008), hypolipidemic, hypocholesterolemic (Berrougui et al. 2003), inhibits platelet aggregation (Mekhfi et al. 2008), antithrombotic (Mekhfi et al. 2012), and prevents cancer (Khallouki et al. 2003). Additionally, several studies have demonstrated that Argan oil has a significant antidiabetic activity (Bouyahya et al. 2021). Previous work reported mechanisms underlying Argania's antidiabetic effect, including inhibition of certain digestive enzymes involved in diabetes, reduced intestinal absorption of D‐glucose, increased peripheral glucose utilization, and potential antioxidant effects (Sabir et al. 2025; Daoudi et al. 2020, 2023). Despite the promising properties of Argan oil, few studies have specifically evaluated its effects on diabetic complications. Moreover, Argan oil can be obtained from either roasted or non‐roasted kernels, and the roasting process is known to influence its chemical composition, particularly phenolic compounds, tocopherols, and antioxidant capacity, which may affect its biological properties (Demnati et al. 2020; Daoudi, Houmy, et al. 2025). The current study primarily focuses on in vivo investigations to evaluate, for the first instance and in a pioneering manner, the hepatoprotective and potential cardioprotective properties of Argan oil against diabetes‐related complications. Notably, this study examines the role of seed roasting in modulating the oil's effects on lipid metabolism and oxidative stress in a streptozotocin‐induced diabetic model.

## 2 Material and Methods

### 2.1 Botanical Sample Collection and Oil Preparation

The fruits of *Argania spinosa* L. (Sapotaceae) were harvested in February 2019 from the Agadir region. A botanist identified the plant with the reference number HUMPOM431. After the Argan fruits were peeled, the seeds were either used directly to produce unroasted Argan oil (HGANT) or roasted, at 100°C for about 20 min, with continuous mixing to ensure homogeneous roasting and prevent localized overheating of the finely sized kernels to produce roasted Argan oil (HGAT). Oil extraction was carried out using a mechanical screw press (cold‐press expeller) operating without the use of solvents, following the same extraction procedure previously reported elsewhere (Daoudi, Mamri, et al. 2025; Daoudi, Houmy, et al. 2025). The extracted oils were filtered to remove solid residues and stored in opaque glass bottles at 4°C until further analysis. The respective yields for HGAT and HGANT were 28.49% and 12.50%.

### 2.2 GC/MS Analysis

The chemical composition of the extract used in this study has already been characterized by gas chromatography coupled with mass spectrometry (GC/MS) in a previous publication (Daoudi, Mamri, et al. 2025). The analysis was performed after methylation of fatty acids following the NF T60‐233 standard, using a SHIMADZU GC/MS‐QP2010 system equipped with a nonpolar column. Compound identification was based on comparison of retention times, mass spectra, and retention indices with authentic standards and the NIST library. In the present study, previously published data are cited to document the chemical composition of the extract.

### 2.3 Animal Model and Diabetes Induction

Adult *Wistar* rats were sourced from the accredited animal house at the Faculty of Sciences, Oujda, Morocco. The animals were maintained under standard laboratory conditions: ambient temperature set at 23°C ± 2°C, a 12‐h light/dark photoperiod, and unrestricted access to both food and water. Prior to the experimental procedures, a 1‐week acclimatization period was observed to allow physiological adaptation. Experimental diabetes was induced using a previously validated protocol (Daoudi et al. 2020). All procedures complied with the ethical guidelines for the care and use of laboratory animals as outlined by the US National Institutes of Health (2015). Only animals that developed clear symptoms of diabetes after induction were included in the study. Animals that did not show diabetic symptoms were excluded.

### 2.4 Experimental Procedure

This work investigated the impact of a 6‐week daily oral administration of HGAT and HGANT (2 mL/kg body weight/day) on cardiac and hepatic function in diabetic rats. The animals were randomly divided into five experimental groups, with six animals per group: a control group and diabetic group receiving distilled water, a positive control group treated with metformin (250 mg/kg body weight/day), an HGAT‐treated group (2 mL/kg body weight/day), and an HGANT‐treated group (2 mL/kg body weight/day). The selected doses of HGAT and HGANT were based on previous studies reporting biological effects of Argan oil following oral administration (Daoudi, Mamri, et al. 2025; Mobashar et al. 2025; Bouhrim et al. 2018; Daoudi et al. 2021). Metformin was used as a reference treatment at a dose of 250 mg/kg body weight/day based on its established use in experimental diabetic rat models (Mobashar et al. 2025). All treatments were administered once daily by oral gavage for 6 weeks.

The sample size (*N* = 30) was determined based on previous similar experimental studies and practical considerations, ensuring sufficient statistical power to detect biologically relevant differences while minimizing the number of animals used. At the end of the experiment, specimens from blood, liver, and heart were gathered and processed according to a previously established protocol. Notably, all animals were anesthetized with isoflurane and then sacrificed to collect blood, liver, and heart specimens, which were processed for biochemical and histopathological analyses according to the previously established protocol (Mobashar et al. 2025).

Comparisons were performed between the normal control and diabetic control groups to confirm successful induction of diabetes. Treated groups (HGAT, HGANT, and metformin) were compared with the diabetic control group to evaluate therapeutic effects. Additionally, treated groups were compared with the normal control group to assess the extent to which treatments restored parameters toward normal physiological values. Comparisons were also conducted among treated groups.

### 2.5 Biochemical Assays

ALT, AST, LDH, Direct and total bilirubin, serum albumin, triglycerides, cholesterol, HDL‐c, LDL‐c, and VLDL‐c were measured using the ARCHITECT ci4100 Analyzer.

### 2.6 Hepatic Glycogen

The hepatic glycogen dosage is performed following previously established protocol (Bouhrim et al. 2018), based on the method described by Ong and Khoo (2000). The preparation of the liver homogenate requires grinding 300–500 mg of liver tissue in 2000 μL of 30% KOH solution. Then, the tissue homogenate was heated to 100°C for 30 min. Four milliliter of 95% ethanol was added to the mixture, which was then kept at 4°C for 30 min. This step is performed twice in succession to precipitate the majority of the glycogen in the homogenate. At the end, the purified glycogen was solubilized in 1000 μL of distilled water, and glycogen content was assessed by adding 5 mL of anthrone reagent. Absorbance was measured at 625 nm, and the results were calculated from the glycogen calibration curve then expressed as mg/g of tissue.

### 2.7 Antioxidant Markers Determination in Heart and Liver Samples

The analysis of antioxidant markers in liver and heart tissues was performed using three key protocols. The level of MDA, a marker of lipid peroxidation, was assessed using the method of Draper and Hadley (1990), where the reaction mixture was heated with TBA, and absorbance was measured at 535 nm. CAT activity, an important antioxidant enzyme, was determined according to the protocol of Aebi (1984), in which hydrogen peroxide degradation was monitored at 240 nm. Lastly, GSH levels, a key indicator of cellular antioxidant capacity, were measured using the Ellman method, with absorbance read at 412 nm after reaction with DTNB (Ellman 1959). The MDA content was expressed as nmol/g tissue, CAT activity was expressed as H_2_O_2_/min/mg protein, and GSH content was expressed as mg/g protein.

### 2.8 Histopathological Analysis

Liver and heart tissues were excised and preserved in buffered formalin solution (10%) for 48 h. Following fixation, the tissues were embedded in paraffin and sectioned at 7 μm using a Leica RM2235 rotary microtome. For histological analysis, the sections were stained using hematoxylin–eosin and Masson's trichrome. Collagen area in the tissue samples was quantified using the “MESURIM_PRO” software (Mamri et al. 2022).

### 2.9 Data Analysis

GraphPad Prism 5 was used for statistical analysis, and results were presented as mean ± standard error of the mean (SEM). Data distribution was assessed using the Shapiro–Wilk test and graphical inspection. The data were considered approximately normally distributed when no significant deviation from normality was detected (*p* > 0.05). Given the small sample size (*n* = 6 per group), the normality results were interpreted cautiously, given the limited power of the test. Comparisons between groups were performed using one‐way analysis of variance (ANOVA) followed by Tukey's post hoc test to identify significant differences between specific groups. A *p* value of < 0.05 was considered statistically significant.

## 3 Results

### 3.1 GC/MS Analysis

The GC/MS analysis previously published in earlier work (Daoudi, Mamri, et al. 2025) revealed that the extract contains four major fatty acids: palmitic acid, oleic acid, linoleic acid, and stearic acid, which were detected in both samples with comparable proportions. A specific additional compound, heptadecane 2,6‐dimethyl‐, was identified only in the roasted oil. Overall, the chemical profile showed a predominance of unsaturated fatty acids, reflected by a higher UFA/SFA ratio, particularly in the unroasted oil, highlighting the nutritional relevance of the extract.

### 3.2 Effect of Argan Oil on Glycemia and Relative Weights of Organs

Table 1 presents the effect of HGAT and HGANT treatments on blood glucose levels in STZ‐diabetic rats before and after intervention. Along with the relative weights of the liver and heart at the end of the experimental period, these findings indicate that administering HGAT and HGANT at 2 mL/kg body weight/day significantly lowered blood glucose levels in STZ‐diabetic rats. By the end of the experimental phase, glycemia levels were significantly reduced following treatment with HGAT and HGANT and approached those observed in the normal control group. Statistical analysis revealed that the glycemia levels in the HGAT and HGANT‐treated groups were in the same range as those observed in the Metformin group, suggesting that Argan oil may be a promising alternative or adjunct to conventional antidiabetic therapies. The findings also revealed a significant increase in relative liver weight in diabetic rats compared with normal rats (*p* < 0.05). Nevertheless, no significant difference in heart weight was observed between the diabetic and normal groups (*p* > 0.05). Treatment with 2 mL/kg body weight/day of HGAT and HGANT resulted in a notable reduction in liver weight in diabetic rats (*p* < 0.01 for HGAT and *p* < 0.05 for HGANT), while heart weight showed no change compared to the diabetic group. Additionally, no significant difference in liver weight was observed between the normal group and the HGAT‐ and HGANT‐treated groups (*p* > 0.05).

**TABLE 1:** Impact of oral administration of HGAT, HGANT, and metformin on glycemia levels before and after treatment, as well as relative liver and heart weights in diabetic *Wistar* rats at the end of treatment.

Groups	Glycemia level (g/L)		Liver (g/kg)	Heart (g/kg)
	Before	After		
Normal	1.04 ± 0.02	0.94 ± 0.04	31.04 ± 2.16	3.39 ± 0.27
Diabetic	4.30 ± 0.28	5.9 ± 0.07	45.96 ± 4.05^a^	4.8 ± 0.6 ns
Diabetic + metformin	4.10 ± 0.04	1.60 ± 0.06	32.48 ± 1.56^b^	3.28 ± 0.11 ns
Diabetic + HGAT	4.73 ± 0.42	1.07 ± 0.04	33.39 ± 2.26^bb^	3.63 ± 0.10 ns
Diabetic + HGANT	3.88 ± 0.57	1.09 ± 0.09	32.2 ± 1.29^b^	3.70 ± 0.22 ns

*Note:* ^a^ *p* < 0.05 compared to the normal group. ^b^ *p* < 0.05; ^bb^ *p* < 0.01 in comparison to the non‐treated diabetic group.

Abbreviation: ns, not significant.

### 3.3 Impact of Argan Oil on Lipid Profile

#### 3.3.1 Impact of Argan Oil on Triglyceride and Total Cholesterol Levels

In this experiment, the impact of HGAT and HGANT on liver metabolic function was assessed by measuring triglyceride and total cholesterol levels in each group (Figure 1). After 6 weeks of intervention, the untreated diabetic group showed a marked elevation in both triglycerides and total cholesterol compared with the normal group (*p* < 0.001 for triglycerides and *p* < 0.01 for total cholesterol). However, 2 mL/kg body weight/day of HGAT and HGANT administered to diabetic rats significantly reduced triglyceride levels (*p* < 0.01 for both HGAT and HGANT) and total cholesterol (*p* < 0.05 for both HGAT and HGANT) when compared to the untreated diabetic group. Despite these reductions, triglyceride levels did not reach those of the normal group, as determined by statistical analysis, whereas total cholesterol levels were comparable to those of the normal group. Metformin treatment led to a significant reduction in both triglyceride levels (*p* < 0.001) and total cholesterol (*p* < 0.05), bringing these values in line with those of the normal group. Statistical comparison between the HGAT and HGANT groups revealed no significant differences in triglyceride or total cholesterol levels (*p* > 0.05), indicating that roasting did not significantly affect these lipid parameters.

**FIGURE 1:**
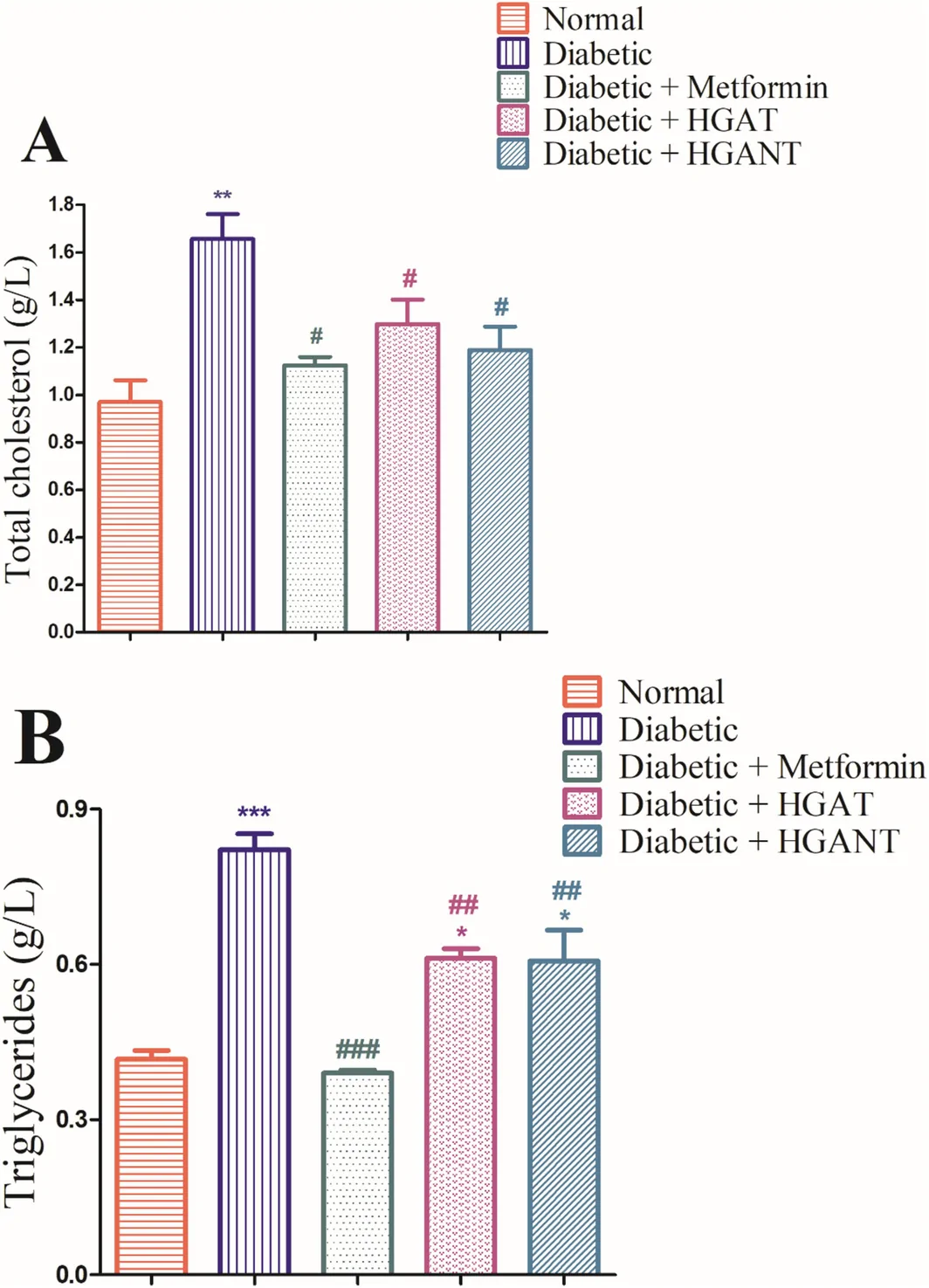
Effect of HGAT, HGANT, and metformin on total cholesterol (A) and triglycerides (B) levels in diabetic rats (*n* = 6). *p < 0.05; **p < 0.01; ***p < 0.001 in comparison to the normal group. ^#^ *p* < 0.05; ^##^ *p* < 0.01; ^###^ *p* < 0.001 in comparison to the non‐treated diabetic group.

#### 3.3.2 Argan Oil Impact on Plasma Lipoproteins LDL‐c, HDL‐c, and VLDL‐c

The effect of HGAT and HGANT on plasma lipoprotein levels is shown in Figure 2. In the diabetic rat group, LDL‐c and VLDL‐c levels were significantly elevated compared to the normal group (*p* < 0.01 for LDL‐c and *p* < 0.001 for VLDL‐c). Conversely, HDL levels showed a substantial reduction compared with the normal group (*p* < 0.05). After 6 weeks of treatment with 2 mL/kg body weight/day of HGAT and HGANT in diabetic rats, there was a significant increase in HDL‐c levels in comparison with the untreated diabetic group (*p* < 0.05 for both HGAT and HGANT). Moreover, statistical analysis showed no significant difference in HDL‐c levels between rats treated with HGAT or HGANT and the normal control group (*p* > 0.05), indicating that HDL‐c levels approached those observed in the normal group. Treatment with HGAT and HGANT for 6 weeks also resulted in significant reductions in LDL‐c (*p* < 0.05 for both HGAT and HGANT) and VLDL‐c (*p* < 0.05 for both HGAT and HGANT) compared with the non‐treated diabetic group. Additionally, metformin treatment significantly reduced LDL‐c (*p* < 0.01) and VLDL‐c (*p* < 0.05) compared with diabetic animals. Based on the results, we can confirm that roasting had no significant effect on the lipid profile.

**FIGURE 2:**
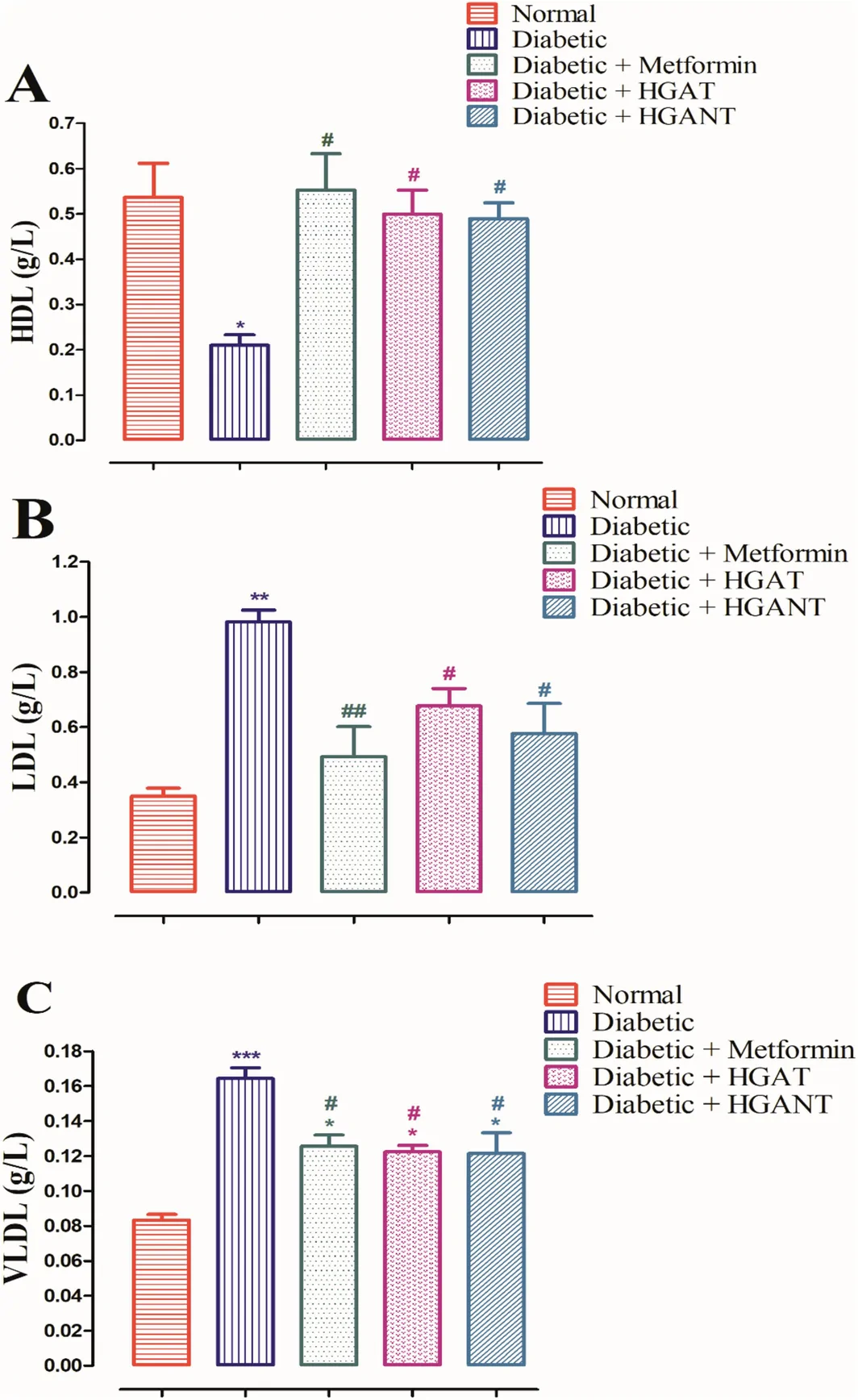
Effect of HGAT, HGANT, and metformin on HDL (A), LDL (B), and VLDL (C) levels in diabetic rats. *p < 0.05; **p < 0.01; ***p < 0.001 in comparison to the normal group. ^#^ *p* < 0.05; ^##^ *p* < 0.01; ^###^ *p* < 0.001 in comparison to the non‐treated diabetic group.

### 3.4 Argan Oil's Effect on Markers of Liver Function

The serum levels of ALT, AST, and LDH were measured to assess liver damage induced by streptozotocin (Figure 3). Following 6 weeks of treatment, a significant elevation in these markers was noted in the diabetic group relative to the normal group (*p* < 0.001 for AST and ALT, *p* < 0.05 for LDH). Treatment with HGAT and HGANT at 2 mL/kg body weight/day notably reduced the elevation of these markers in diabetic rats. Specifically, ALT and LDH levels in the HGAT and HGANT groups were comparable to those in the normal group, although AST levels remained elevated in comparison with normal values (*p* < 0.01 for HGAT, *p* < 0.05 for HGANT). Metformin treatment did not exhibit significant differences relative to the non‐treated diabetic group. These findings indicate that no significant differences were observed between HGAT and HGANT for ALT, AST, and LDH levels, suggesting that the roasting process did not influence these parameters.

**FIGURE 3:**
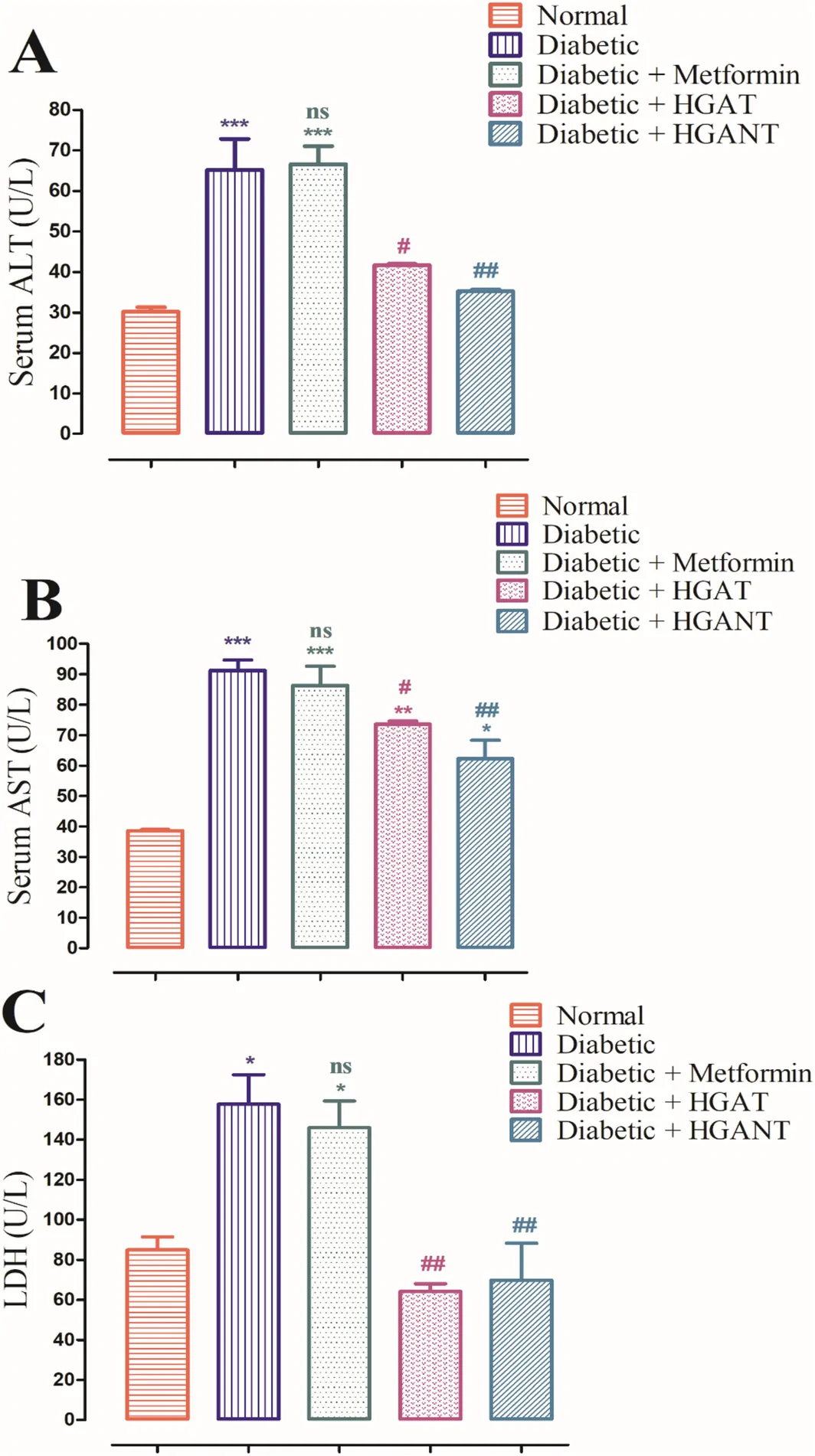
Effect of Argan oil treatment on liver makers levels ALT (A), AST (B), and LDH (C) in *Wistar* rats. *p < 0.05; **p < 0.01; ***p < 0.001 in comparison to the normal control. ^#^ *p* < 0.05; ^##^ *p* < 0.01; ^###^ *p* < 0.001; ns, not significant in comparison to the diabetic control.

### 3.5 Argan Oil Impact on Direct and Total Bilirubin

Figure 4 shows the effect of administering HGAT and HGANT at 2 mL/kg body weight/day on direct (A) and total (B) bilirubin levels in diabetic rats. At the end of 6 weeks, it was observed that direct and total bilirubin levels in diabetic rats significantly increased compared to the normal group (*p* < 0.01 for both parameters). Therefore, an impairment in the excretory function of hepatocytes may be suggested. Treatment with HGAT and HGANT at a dose of 2 mL/kg body weight/day for 6 weeks resulted in a significant reduction in both direct and total bilirubin levels compared to the untreated diabetic group (*p* < 0.01 for HGAT and *p* < 0.001 for HGANT). These findings suggest that HGAT and HGANT may improve hepatic excretory function in streptozotocin‐induced diabetic rats. However, treatment with metformin did not induce any significant changes compared to the untreated diabetic group. These results indicate that roasting Argan seeds did not significantly affect direct and total bilirubin levels.

**FIGURE 4:**
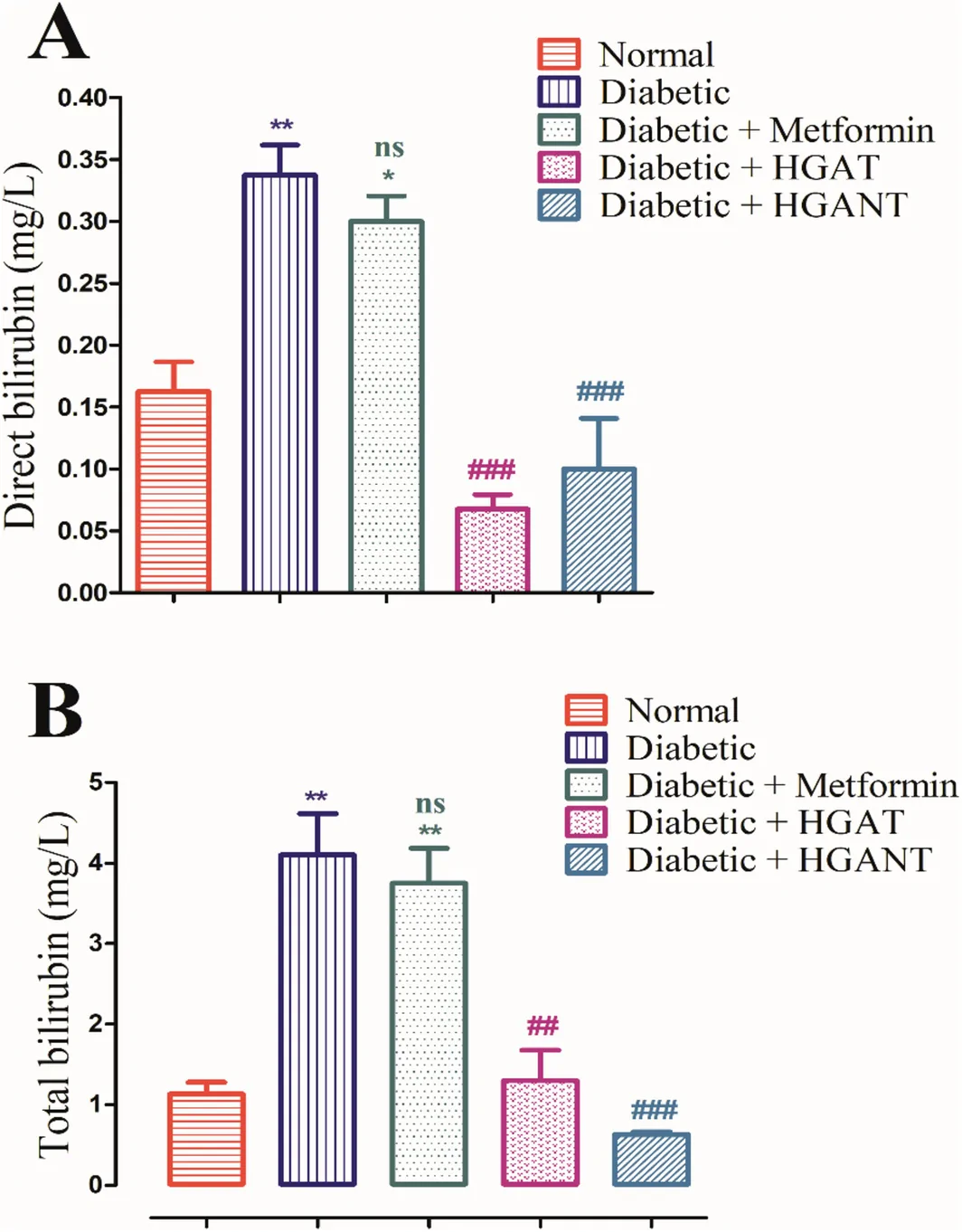
Effect of HGAT, HGANT, and metformin on direct (A) and total (B) bilirubin levels in diabetic rats. *p < 0.05; **p < 0.01 in comparison to the normal group. ^##^ *p* < 0.01; ^###^ *p* < 0.001; ns, not significant in comparison to the non‐treated diabetic group.

### 3.6 Effect of Argan Oil Treatment on Hepatic Glycogen

The action of HGAT and HGANT on the hepatic glycogen content in diabetic rats is illustrated in Figure 5. The findings indicated that the glycogen content in the normal group was 1.01 ± 0.03 mg/g of tissue. In contrast, the diabetic group exhibited a significant decrease in glycogen levels (*p* < 0.01), with a value of 0.64 ± 0.05 mg/g of tissue. Treatment with HGAT, HGANT, and metformin led to a notable increase in hepatic glycogen levels (*p* < 0.01 for HGAT; *p* < 0.05 for HGANT and metformin) compared with the diabetic group, with values statistically comparable to those of the normal group. These results indicate that HGAT had the same effect as HGANT, suggesting that roasting did not influence hepatic glycogen levels.

**FIGURE 5:**
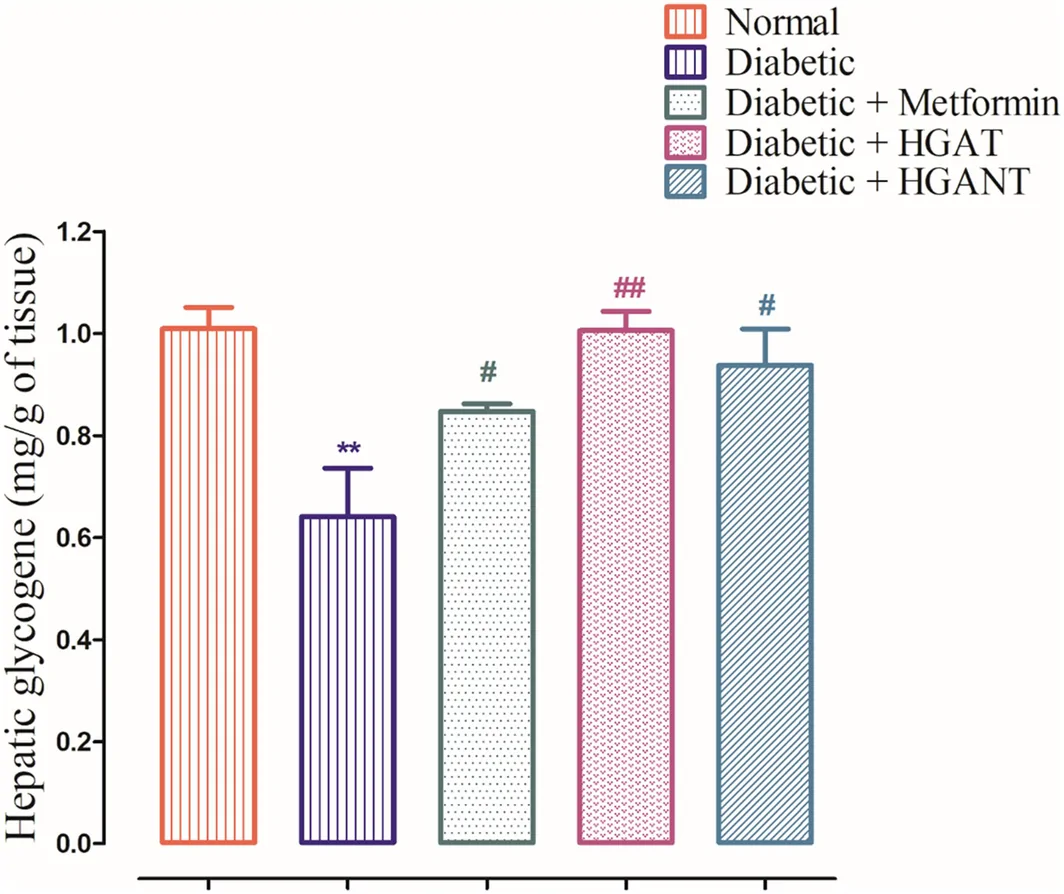
Effect of HGAT, HGANT, and metformin on hepatic glycogen in diabetic rats. **p < 0.01 compared to the normal group. ^#^ *p* < 0.05; ^##^ *p* < 0.01 in comparison to the non‐treated diabetic group.

### 3.7 Argan Oil Treatment Effect on Oxidative Stress Markers

The impact of HGAT and HGANT on oxidative stress markers, including lipid peroxidation (Figure 6), GSH (Figure 7), and CAT (Figure 8) levels, was evaluated in the liver and heart of streptozotocin‐induced diabetic rats. Lipid peroxidation, assessed through MDA levels, was significantly elevated in the liver (*p* < 0.001) and heart (*p* < 0.05) of diabetic rats compared to the normal group. Treatment with HGAT, HGANT, and metformin effectively reduced MDA levels in both organs relative to the diabetic group. Notably, in the heart, MDA levels returned to normal after treatment. However, in the liver, although a significant reduction was observed (*p* < 0.001 for HGAT and HGANT), values remained above normal. Statistical comparisons between HGAT and HGANT revealed that the roasting process had no effect on MDA levels. Diabetes induction led to a marked depletion of GSH in both the liver and heart (*p* < 0.001). Administration of HGAT and HGANT significantly enhanced GSH levels in these organs compared to the diabetic control. In the heart, GSH levels returned to normal with HGAT, HGANT, and metformin treatment. However, in the liver, despite improvement, values remained less than normal for HGAT (*p* < 0.05). Interestingly, metformin treatment did not lead to significant changes in GSH levels in comparison to the non‐treated diabetic animals. Similar to MDA, roasting had no notable effect on GSH levels. Moreover, diabetic rats exhibited a significant reduction in cardiac CAT levels (*p* < 0.001). Treatment with HGAT and HGANT led to a significant improvement, although levels remained below normal despite intervention with metformin. In contrast, hepatic CAT levels in the HGAT‐ and HGANT‐treated groups were restored to normal, whereas metformin treatment had no significant effect. Once again, the roasting process did not appear to affect CAT levels.

**FIGURE 6:**
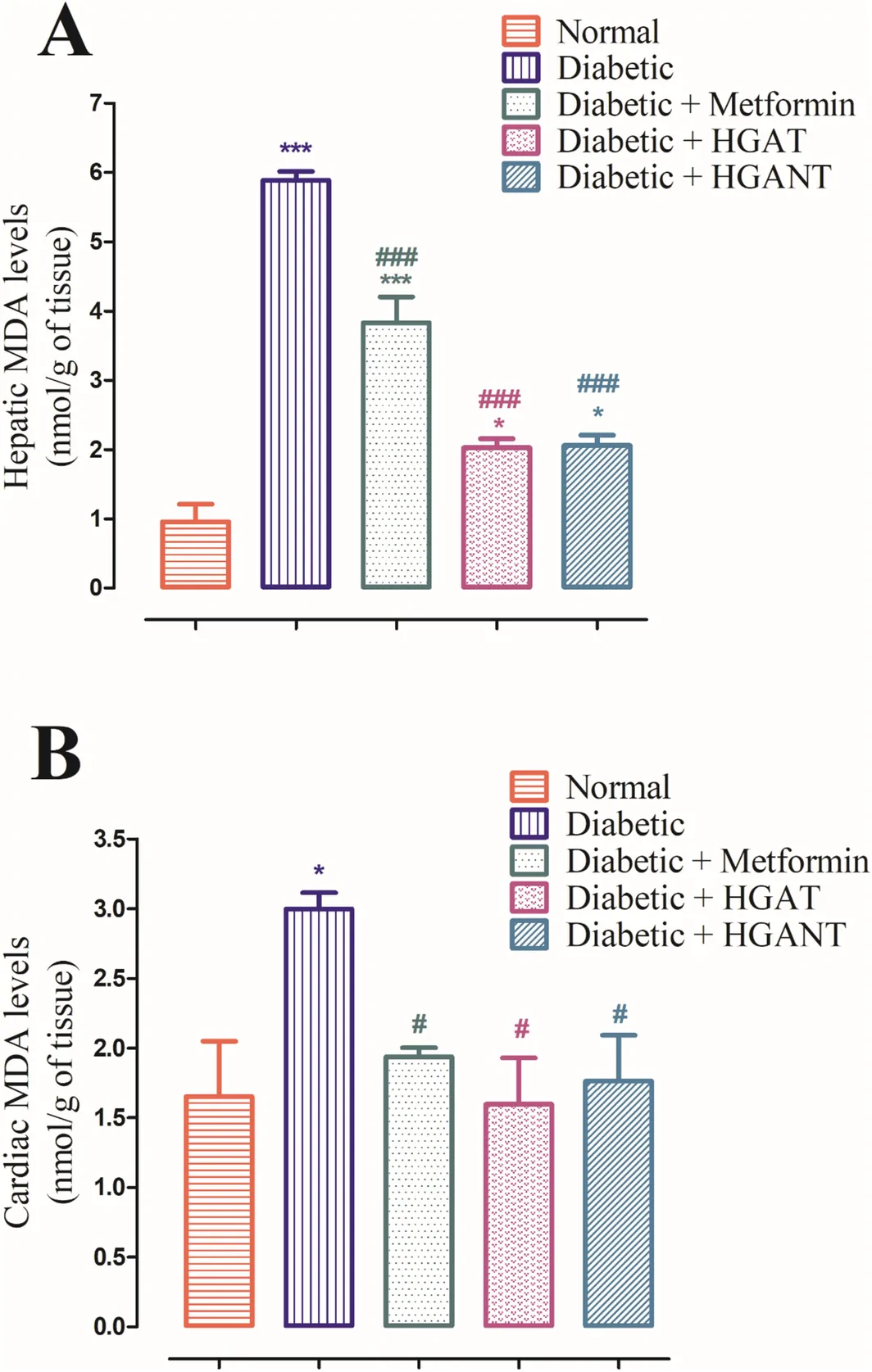
Effect of HGAT, HGANT, and metformin on lipid peroxidation in the liver (A), and heart (B) of diabetic rats. *p < 0.05; **p < 0.01; ***p < 0.001 in comparison to the normal group. ^#^ *p* < 0.05; ^###^ *p* < 0.001 in comparison to the non‐treated diabetic group.

**FIGURE 7:**
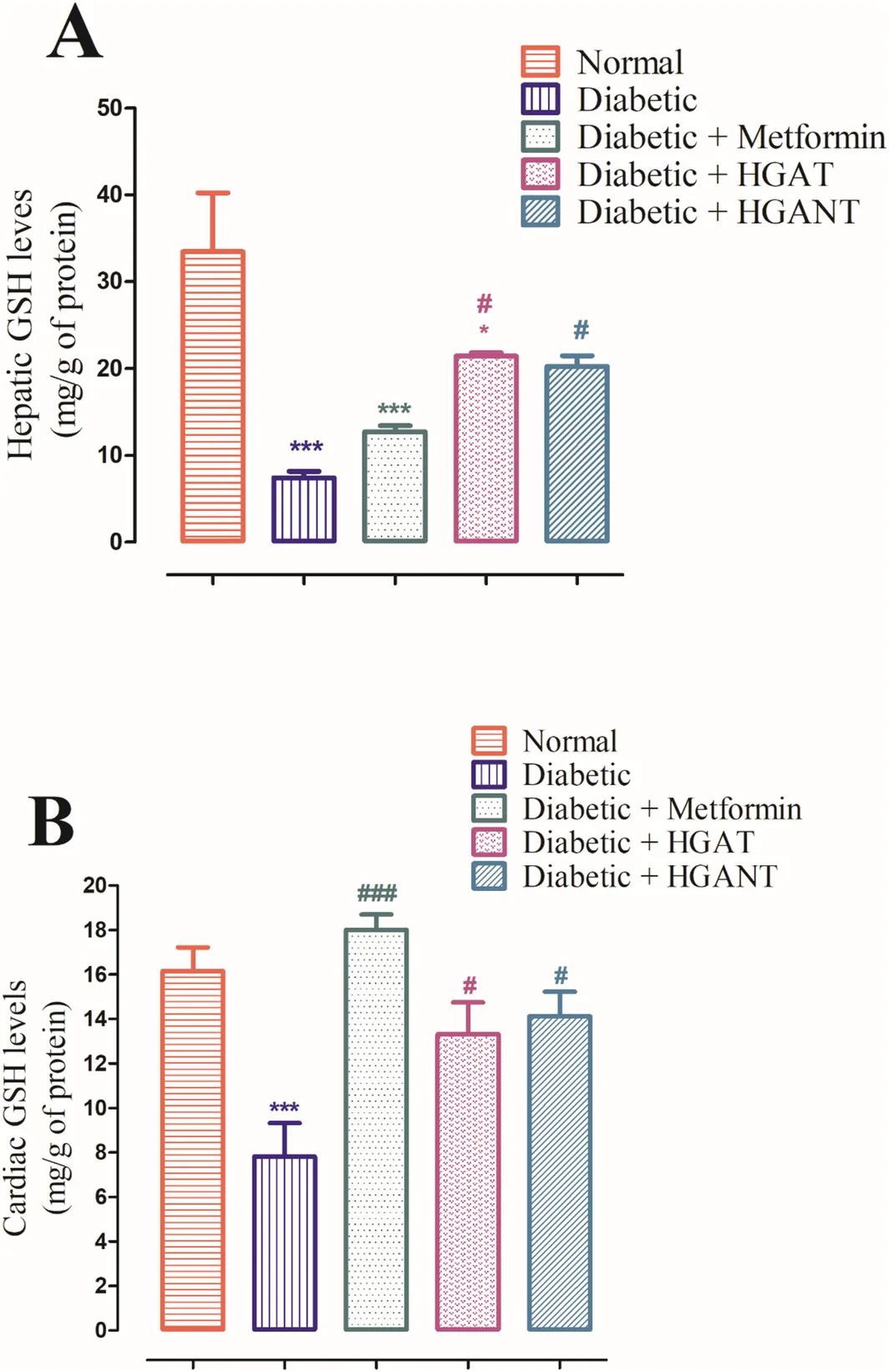
Effect of HGAT, HGANT, and metformin on GSH levels in the liver (A), and heart (B) of diabetic rats. *p < 0.05; ***p < 0.001 in comparison to the normal group. ^#^ *p* < 0.05; ^##^ *p* < 0.01; ^###^ *p* < 0.001 in comparison to the non‐treated diabetic group.

**FIGURE 8:**
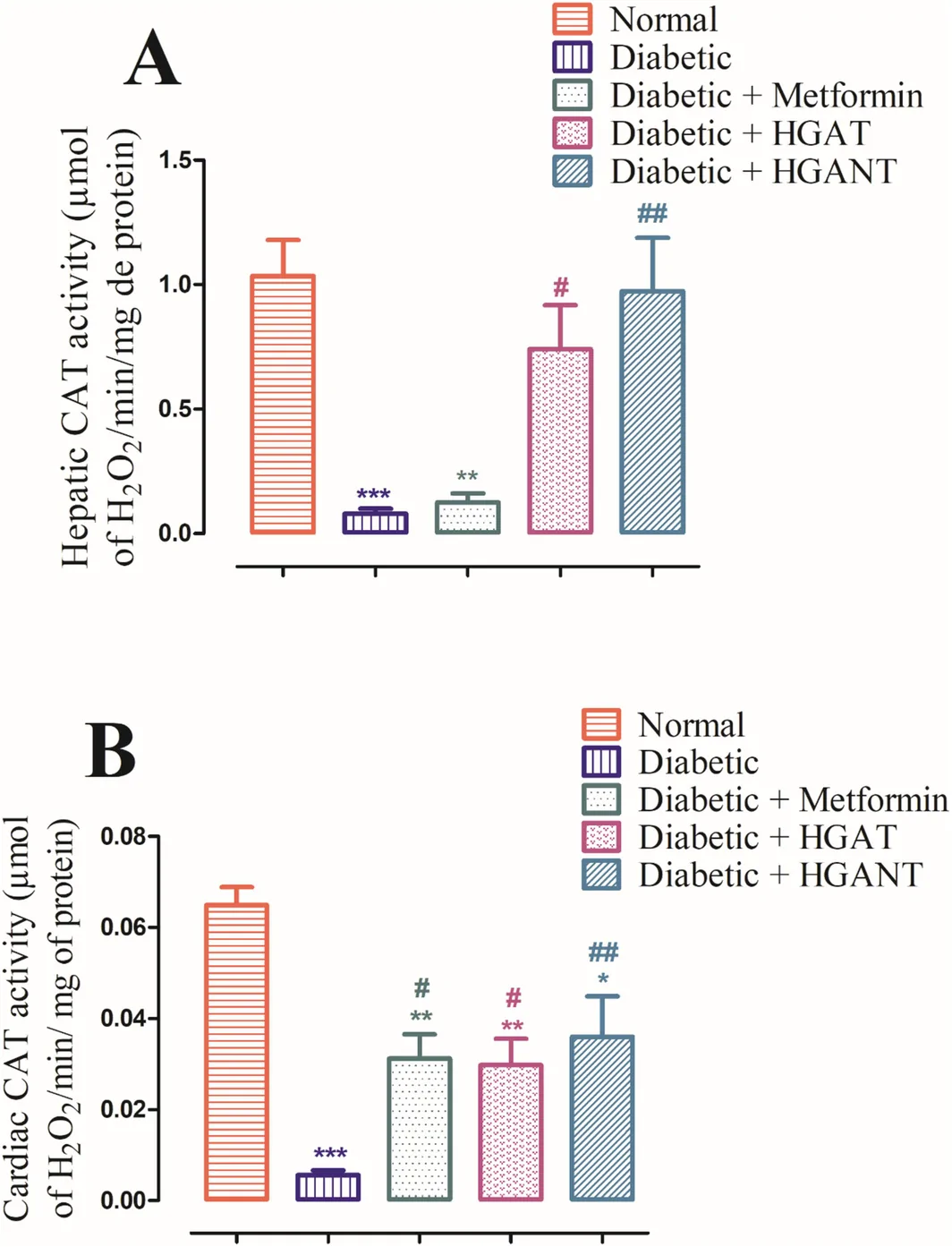
Effect of HGAT, HGANT, and metformin on catalase levels in the liver (A), and heart (B) of diabetic rats. *p < 0.05; **p < 0.01; ***p < 0.001 in comparison to the normal group. ^#^ *p* < 0.05; ^##^ *p* < 0.01; ^###^ *p* < 0.001 in comparison to the non‐treated diabetic group.

### 3.8 Liver Histopathological Analysis

Hematoxylin–eosin staining results obtained during the histological examination of the liver tissues from diabetic rats treated with HGAT, HGANT, and metformin are illustrated in Figure 9. In the normal group, microscopic observation shows that the histoarchitecture of the hepatic cells is normal. In the non‐treated diabetic group, the appearance of lipid droplets with necrosis of the hepatic cells is observed in all rats. Treatment with HGAT or HGANT protected the liver against the appearance of these lipid droplets. Figure 10 presents the results of the Masson's trichrome staining of the liver, which stains collagen blue, the nuclei of hepatocytes dark red inside the cells, while the cytoplasm is stained red. In the diabetic group, microscopic observation shows the presence of significant deposits of collagen fibers in the portal triad compared to the normal group. Additionally, it is observed that the nuclei of hepatocytes exhibited signs of pyknosis. Treatment with HGAT and HGANT significantly prevented the accumulation of collagen fibers and limited damage to the hepatic tissue. In contrast, treatment with metformin induced a reduction in the percentage of collagen in comparison with the diabetic group, but this decline did not reach normal values (Figure 11).

**FIGURE 9:**
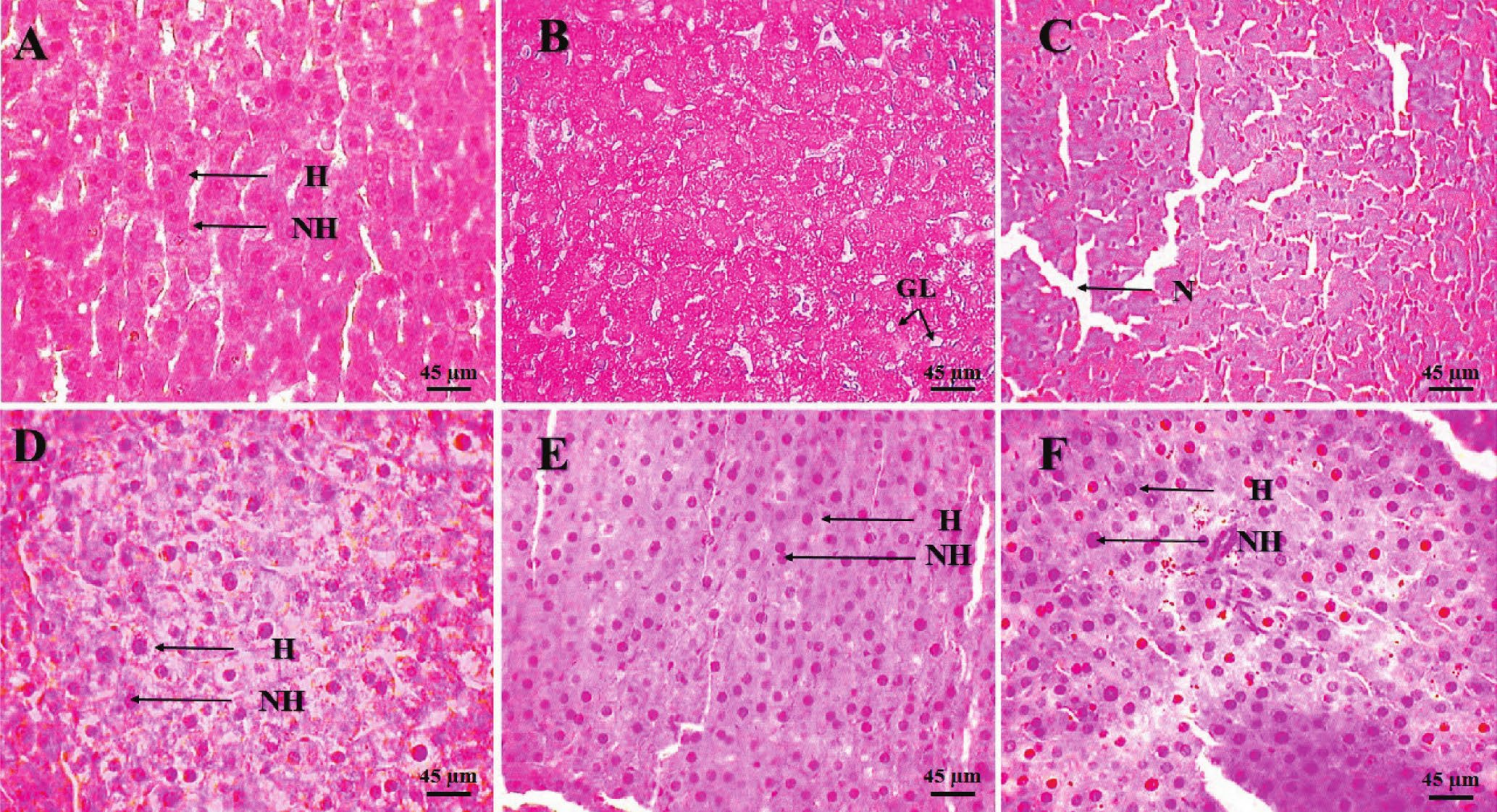
Effect of HGAT, HGANT, and metformin on the histological features of the liver in diabetic rats at the end of treatment. (A) Normal group; (B, C) non‐treated diabetic group; (D) diabetic group treated with metformin; (E) group treated with HGAT; (F) group treated with HGANT. The liver sections were stained with HE and observed under a light microscope (Optika microscopy, Italy) using a 40× objective. GL, lipid droplets; H, hepatocyte; N, hepatic necrosis; NH, nucleus of hepatocytes.

**FIGURE 10:**
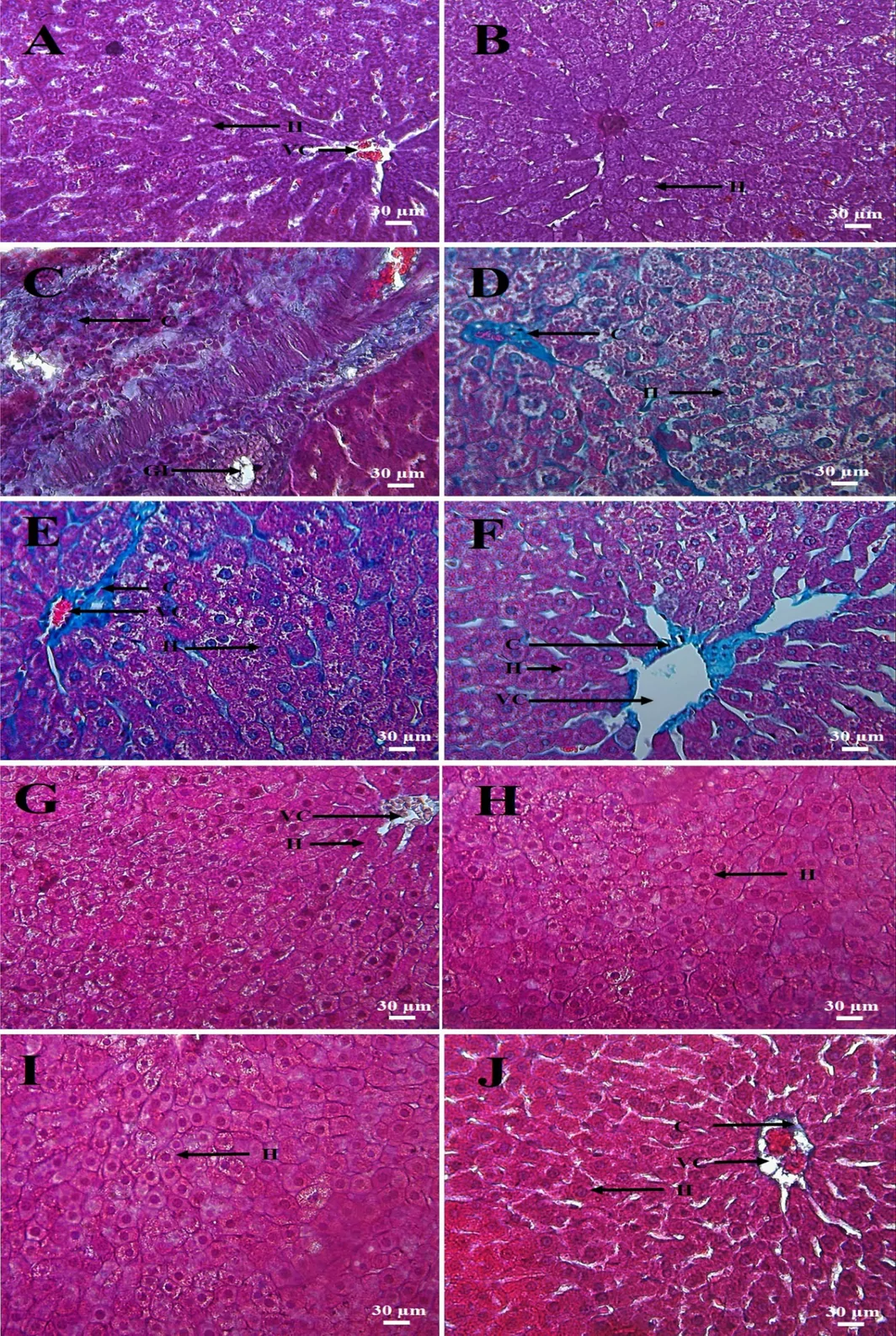
Effect of HGAT, HGANT, and metformin on the histological features of the liver in diabetic rats at the end of treatment. (A, B) Normal group; (C, D) non‐treated diabetic group; (E, F) diabetic group treated with metformin; (G, H) group treated with HGAT; (I, J) Group treated with HGANT. The sections were stained with Masson's Trichrome and observed under a light microscope (Optika microscopy, Italy) using a 40× objective. C, collagen; GL, lipid droplet; H, hepatocyte; VC, central vein.

**FIGURE 11:**
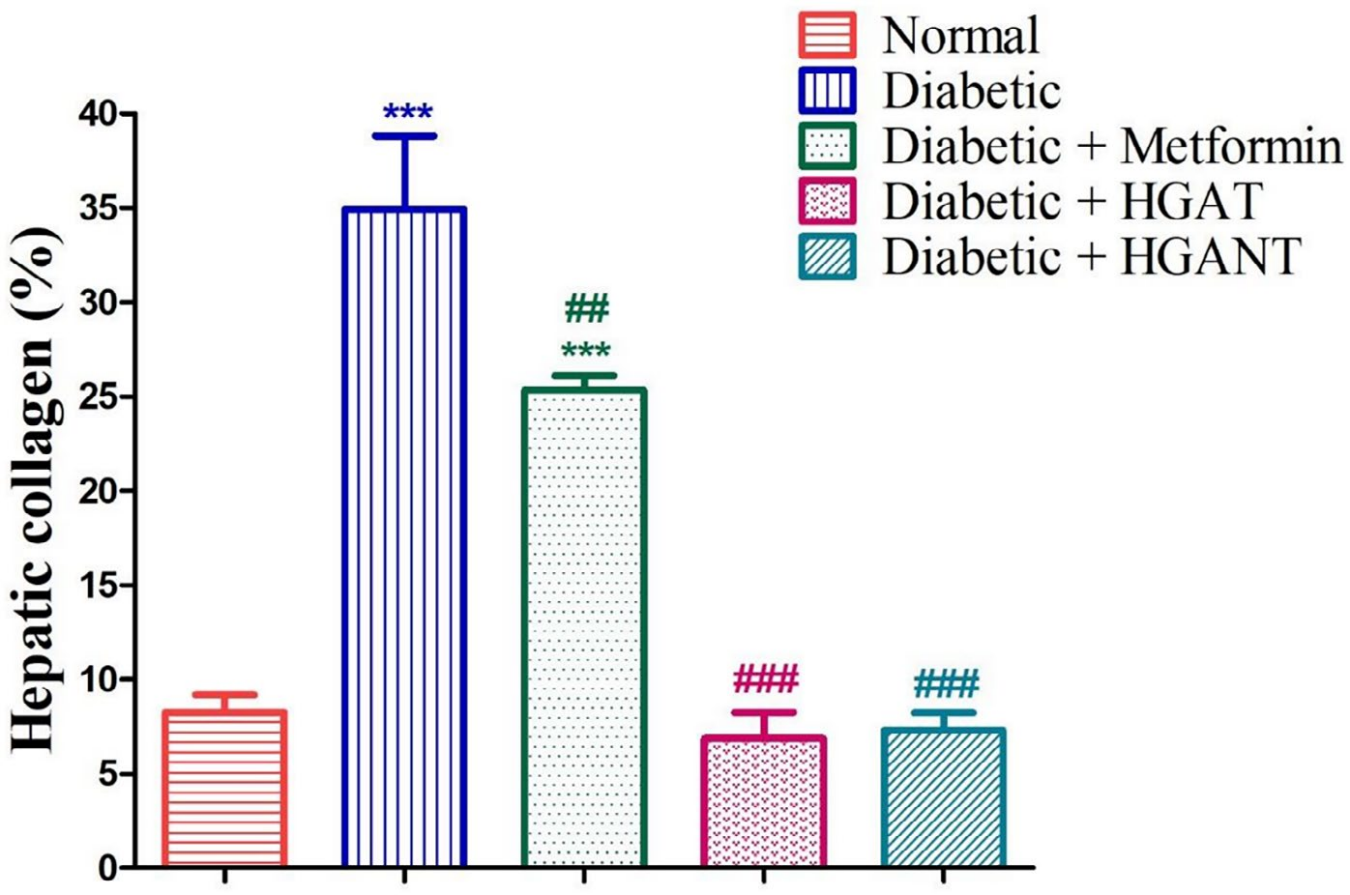
Percentage of hepatic collagen in diabetic groups treated with HGAT, HGANT, and metformin. ***p < 0.001 in comparison to the normal group. ^##^ *p* < 0.01 and ^###^ *p* < 0.001 in comparison to the non‐treated diabetic group.

### 3.9 Histopathology of the Heart

Microscopic observation of cardiac tissue revealed that the myocardial fibers are arranged regularly, and the cardiomyocytes exhibit a normal morphology with distinct cell borders in the animals of the normal control group (Figure 12A). However, in the untreated diabetic group (Figure 12B), the arrangement of cardiac fibers is disrupted. Additionally, there are alterations in the nuclei of some cardiomyocytes, along with an increased volume of cell borders. Treatment with 2 mL/kg body weight/day of HGAT or HGANT in diabetic animals significantly improved the structural abnormalities in cardiac tissue. Masson's trichrome staining of cardiac tissues (Figure 13) shows that in normal rats, the myocardial tissue exhibits normal morphology. In the diabetic group, there are significant deposits of collagen fibers in the myocardium in comparison with the normal group, leading to the development of cardiac fibrosis. Treatment with HGAT, HGANT, and metformin significantly reduced the appearance of collagen compared to the diabetic group (Figure 14) and prevented cardiac damage.

**FIGURE 12:**
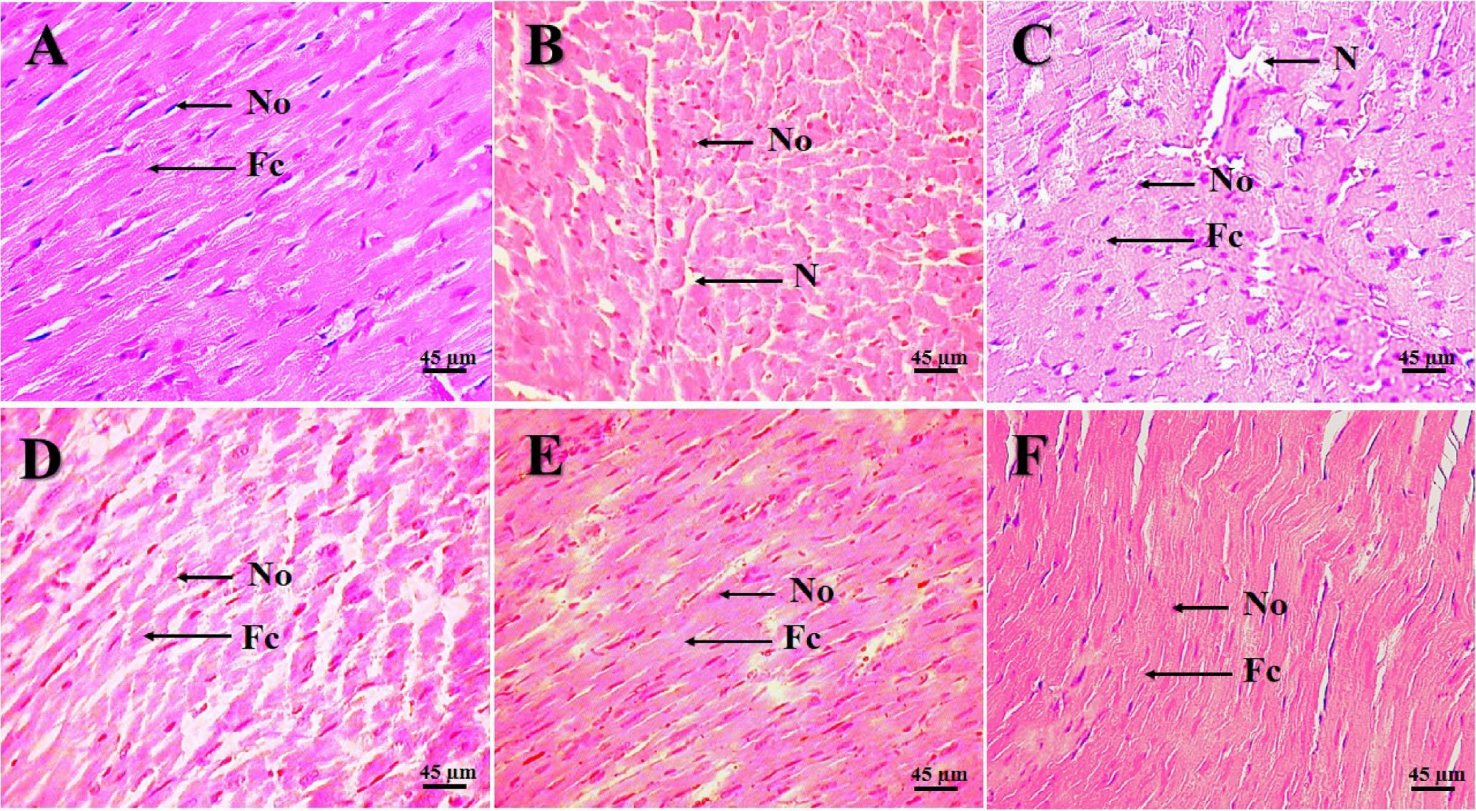
Effect of oral administration of HGAT, HGANT, and metformin on the histological features of the heart in diabetic rats at the end of the treatment. (A) Normal group; (B, C) non‐treated diabetic group; (D) diabetic group treated with metformin; (E) group treated with HGAT; (F) group treated with HGANT. The heart sections were stained with HE and observed under an optical microscope (Optika microscopy, Italy) using a 40× objective. FC, cardiac fibers; N, cardiac necrosis; No, nuclei.

**FIGURE 13:**
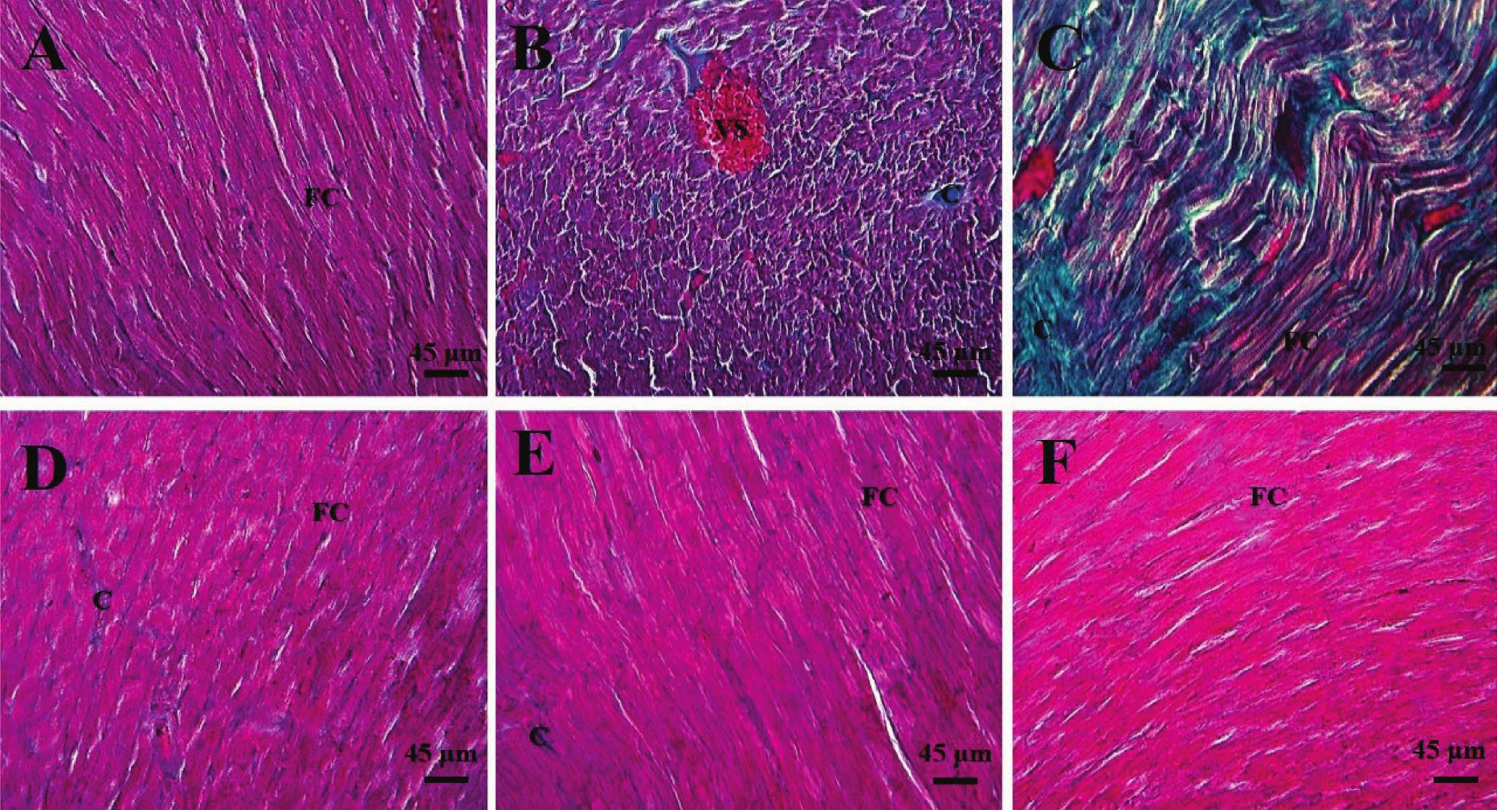
Effect of oral administration of HGAT, HGANT, and metformin on the histological features of the heart in diabetic rats at the end of the treatment. (A) Normal group; (B, C) non‐treated diabetic group; (D) diabetic group treated with metformin; (E) group treated with HGAT; (F) group treated with HGANT. The heart sections were stained with Masson's trichrome and observed under an optical microscope (Optika microscopy, Italy) using a 40× objective. C, collagen; FC, cardiac fiber; VS, blood vessel.

**FIGURE 14:**
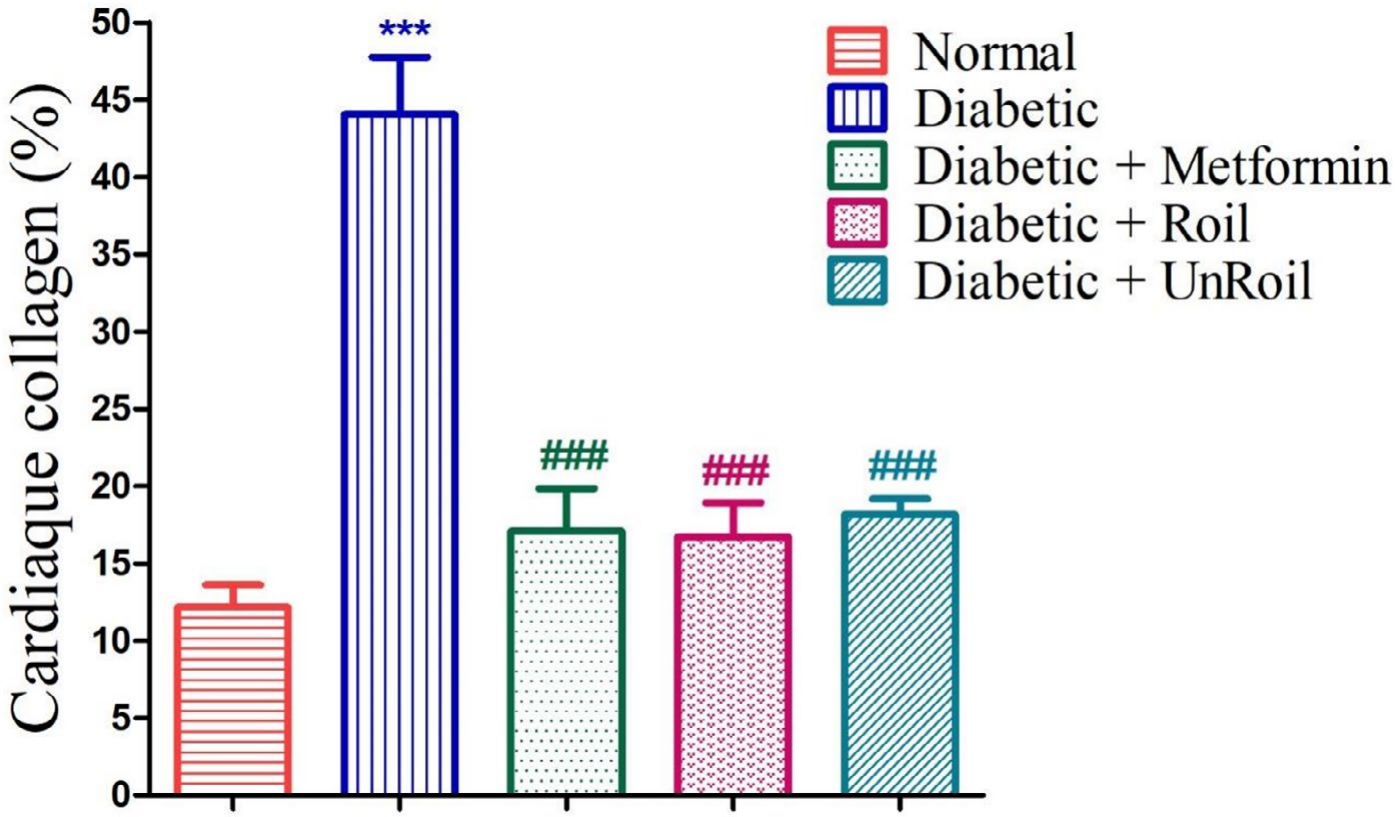
Percentage of cardiac collagen in diabetic groups treated with HGAT, HGANT, and metformin. ***p < 0.001 in comparison to the normal group; ^###^ *p* < 0.001 in comparison to the non‐treated diabetic group.

## 4 Discussion

The ongoing research focuses on the protective effects of Argan oil in managing diabetes‐related complications, with particular emphasis on cardiac and hepatic health. Argan oil, known for its rich composition of bioactive compounds, has shown promising results in improving health (Daoudi et al. 2023). In addition to the current focus on the cardioprotective and hepatoprotective effects of Argan oil on complications associated with diabetes, a previous study also assessed its impact on diabetic nephropathy. The findings indicated that Argan oil may have a protective role in preventing diabetic nephropathy, highlighting its broader protective potential in managing diabetes‐related organ complications (Mobashar et al. 2025).

In this research, the experimental model is based on streptozotocin, a substance isolated from the bacterium *Streptomyces achromogenes* , which is known for its Gram‐positive properties. Currently, this model is the most widely used in animals to induce diabetes, its cytotoxic effects, and its selective action on pancreatic β cells. Indeed, streptozotocin functions as an alkylating agent, which interferes with DNA, leading to reduced insulin secretion and synthesis, and ultimately inducing apoptosis in pancreatic β‐cells (Annadurai et al. 2012). This work showed that intraperitoneal administration of streptozotocin in rats leads to a significant increase in blood glucose levels and a substantial decrease in body weight, both commonly associated with diabetes. After 6 weeks of treatment with roasted and unroasted Argan oil, these adverse effects were significantly reduced, and growth parameters showed noticeable improvement in the diabetic rats (Mobashar et al. 2025). Moreover, it has been documented that streptozotocin also causes hepatotoxicity (Ohaeri 2001). This effect was also observed in the present study, as reflected by increased levels of ALT, LDH, AST, and direct and total bilirubin. In fact, it has been reported that the plasma concentrations of ALT, AST, and ALP are the main markers of liver injury (Anand et al. 1992), and that elevated levels of total and direct bilirubin are associated with the occurrence of hepatocellular problems, obstruction of bile excretion into the duodenum, hemolysis, and defects in the hepatic uptake of bilirubin (Bigoniya and Rana 2010). The increase in the levels of hepatic enzymes in plasma is attributed to liver damage, as they are of cytoplasmic origin and are released into the blood following liver injury (Sharma and Shukla 2011). Both roasted and unroasted Argan oil can protect the liver against damage induced by streptozotocin by decreasing the values of hepatic parameters (ALT, AST, LDH, direct and total bilirubin). Moreover, streptozotocin causes an elevation in plasma triglycerides, total cholesterol, LDL‐c, and VLDL‐c levels, along with a reduction in HDL‐c levels (Howard et al. 2000). The buildup of triglycerides and LDL‐c in hepatocytes results in the development of hepatic steatosis (l‐Mehdar et al. 2015). After 6 weeks of treatment with both roasted and unroasted Argan oil, these parameters significantly improved and approached the values observed in the normal control group, which may be related to the substantial concentration of phytosterols in Argan oil (Moghadasian et al. 1999; El Kharrassi et al. 2014). In fact, phytosterols are crucial in decreasing cholesterol absorption by competing with cholesterol for incorporation into mixed micelles, thereby limiting its uptake through the intestinal barrier (Calpe‐Berdiel et al. 2005; Poli et al. 2021). In addition, the high content of unsaturated fatty acids in Argan oil may contribute to the improvement of lipid metabolism by modulating hepatic lipid metabolism and lipid oxidation (El Kebbaj et al. 2024). The antioxidant properties of Argan oil may also contribute indirectly to the improvement of the lipid profile by attenuating oxidative stress associated with diabetes (Rabbaa et al. 2025; Ould Mohamedou et al. 2011). These complementary mechanisms may partly explain the reductions in triglyceride and total cholesterol levels observed following HGAT and HGANT treatment. Consequently, the improvement of the lipid profile following Argan oil treatment may contribute to the prevention or attenuation of diabetes‐related complications. Hepatic glycogen serves as a stored form of glucose in the liver, reflecting the activation of glycogen synthase and the suppression of glycogen phosphorylase due to the effects of insulin (Vats et al. 2004). In diabetic rats, it is observed that streptozotocin caused a notable decrease in hepatic glycogen, which could be attributed to insulin deficiency (Bollen et al. 1998). However, at the end of the treatment, the amount of hepatic glycogen showed a significant increase, reaching normal levels, thanks to the treatment with Argan oil. The restoration of normal hepatic glycogen levels through both roasted and unroasted Argan oil is likely due to the activation of cells to release insulin, which would activate the process of glycogenesis (Kamalakkannan and Prince 2005).

Lipid peroxidation is a major marker of oxidative stress, contributing significantly to the oxidative degradation of polyunsaturated fatty acids in membrane phospholipids, which results in the formation of free radicals like 4‐hydroxy‐2‐nonenal and MDA (Devbhuti et al. 2009; Cheng et al. 2013). MDA is widely regarded as a key indicator of lipid peroxidation. In the current intervention, an increase in MDA levels was observed in the kidneys, liver, and heart of rats injected with streptozotocin. Nevertheless, daily treatment for 6 weeks with both HGAT and HGANT significantly reduced lipid peroxidation in these organs. These findings indicate that Argan oil could reduce lipid peroxidation in diabetic rats by suppressing the production of reactive oxygen species triggered by streptozotocin, thus decreasing oxidative stress. Additionally, CAT plays a crucial role in converting H_2_O_2_ into water and oxygen, thereby protecting organs and tissues from the damaging effects of free radicals (Naik and Panda 2007). In this investigation, streptozotocin significantly reduced GSH and CAT levels compared with the normal group, suggesting increased free radical formation and depletion of antioxidant defenses. Treatment with HGAT and HGANT improved these levels significantly. Similarly, it was determined previously that the administration of roasted and unroasted Argan oils has hepatoprotective activity. Both oils can protect organs against damage caused by CCl_4_ by decreasing the levels of total/direct bilirubin, AST, ALP, ALT, and MDA. The intraperitoneal injection of CCl_4_ led to a marked rise in direct bilirubin, total bilirubin, triglycerides, LDL‐c, MDA, and the liver weight ratio. On the other hand, it notably decreased hepatic glycogen, HDL‐c, GSH, and body weight gain compared to the control group. Furthermore, CCl_4_ had no impact on total cholesterol levels (Daoudi et al. 2021). The increase in liver weight ratio may be due to lipid and collagen accumulation in liver cells (Villanueva‐Toledo et al. 2020).

Based on the results obtained, Argan oil may be beneficial for diabetics to prevent the onset of diabetic complications due to its beneficial effects on lipid, hepatic, and renal metabolism, as well as its antioxidant capacity, which helps reduce oxidative stress and improve liver, kidney, and heart function. To confirm these findings, a histopathological examination of the cardiac and hepatic tissues was conducted in all treated groups. In the group of diabetic rats, streptozotocin led to structural alterations in both liver and heart tissues, which was attributed to lipid peroxidation. Diabetic cardiomyopathy and hepatopathy are characterized by the accumulation of certain proteins, such as collagen. In the heart, this protein accumulates significantly in the interstitial space, leading to fibrosis and heart failure (Li et al. 2012). These damages are linked to functional and molecular alterations that could decrease ventricular compliance and increase the risk of arrhythmias (Samuel and Delcayre 2017). In the liver, collagen fiber deposition is substantial in periportal and pericentral spaces, reflecting microvascular abnormalities and hepatic fibrosis (Komolkriengkrai et al. 2019). The present study indicates that streptozotocin induces a significant accumulation of collagen fibers in the tissues examined, which explains the increase in organ weight (Villanueva‐Toledo et al. 2020). However, treatment with roasted and unroasted Argan oil showed no significant alterations in the liver or heart and significantly prevented cardiac and hepatic fibrosis compared to the non‐treated diabetic group. Furthermore, the roasting process had no significant impact on the assessed histological and biochemical parameters. This suggests that the biomolecules involved in the improvement of diabetes, as well as the effects of Argan oil on diabetic cardiomyopathy and liver diseases associated with diabetes, remain unaffected by this process. This study aimed to investigate the role of seed roasting in modulating the effects of Argan oil on oxidative stress and lipid metabolism in streptozotocin‐induced diabetic rats. The results showed that roasting did not significantly affect the lipid profile, liver enzyme markers, or oxidative stress markers such as MDA, GSH, and CAT levels. However, the HGAT significantly reduced ALT levels compared to HGANT, suggesting a mild hepatoprotective effect. Additionally, no influence was observed on hepatic glycogen levels or collagen deposition in the liver and heart, indicating that roasting had limited impact on these parameters.

Metformin is an oral medication commonly prescribed for Type 2 diabetes due to its ability to lower blood sugar levels. It works by enhancing insulin sensitivity, promoting glucose uptake in the liver, muscles, and adipose tissues, while also decreasing glucose production in the liver (Biondani and Peyron 2018). The use of metformin at 250 mg/kg body weight/day as a positive control significantly improved metabolic function markers (total cholesterol, triglycerides, LDL‐c, VLDL‐c, HDL‐c, and hepatic glycogen). However, it showed no effect on liver function markers and excretory function indicators (total/direct bilirubin). This lack of significant improvement may be related to the experimental conditions, including the streptozotocin‐induced diabetic model, the dose and duration of metformin treatment, and the extent of hepatic alterations in the diabetic rats. Thus, the absence of a significant effect on these parameters does not necessarily indicate a lack of hepatoprotective potential of metformin, but may reflect the specific experimental conditions of the present study. This drug also has cardioprotective properties in Type 2 diabetics, as it improves cardiac function and reduces cardiomyocyte apoptosis in diabetic mice (Yang et al. 2020). Additionally, it plays a role in treating liver diseases, especially those related to diabetes and inflammation, by reducing oxidative stress in the liver (Bhat et al. 2015). In fact, some studies have reported that metformin does not worsen liver lesions caused by diabetes and, therefore, is not considered hepatotoxic, although it may cause lactic acidosis as a side effect (Brackett 2010). Recently, however, several cases of liver injury have been reported with metformin, mainly reflected by elevated liver enzymes (Volarevic et al. 2015), which is consistent with the results of the present study.

While the present study provides valuable insights into the protective effects of roasted and unroasted Argan oil against diabetes‐related complications, some limitations should be considered. The sample size was relatively limited (*n* = 6 per group), which, although consistent with similar experimental studies, may influence the statistical power of the analysis. In addition, the assessment of data normality was primarily based on visual inspection due to the small sample size, which represents a methodological constraint. Furthermore, the study was conducted using a streptozotocin‐induced diabetic rat model, which, despite being well established, may not fully replicate the complexity of human diabetes. The investigation was also limited to a single dose and a 6‐week treatment period, which does not allow for evaluation of long‐term effects or dose–response relationships. Moreover, although biochemical and histological analyses provided strong evidence of protective effects, further studies exploring the underlying molecular mechanisms would help to better elucidate the pathways involved.

## 5 Conclusion

This work highlights the promising protective effects of Argan oil against diabetes‐related complications, particularly on cardiac and hepatic functions. The outcome of this work demonstrates that both oils improved lipid profiles and liver enzyme levels, with ALT and LDH returning to normal, while AST remained slightly elevated. Oxidative stress markers were partially restored in an organ‐specific manner: in the heart, MDA and GSH returned to normal and CAT levels improved, whereas in the liver, MDA and GSH were significantly reduced but remained above normal, and CAT returned to baseline. Cardiac parameters also improved, suggesting a potential cardioprotective effect. While the roasting process did not affect any of these biological activities, indicating that traditional preparation, which often does not control temperature, preserves the medicinal properties of Argan oil. These findings suggest that Argan oil, whether roasted or unroasted, could serve as an effective complement to existing treatments for preventing and managing diabetes complications, offering new perspectives in the management of this disease. However, several additional studies are recommended to better understand the underlying mechanisms of the protective effects of Argan oil, particularly concerning its long‐term impact on diabetes‐related complications. It would also be relevant to explore its interactions with other antidiabetic treatments, as well as its efficacy in larger clinical models.

## Author Contributions

**Nour Elhouda Daoudi:** conceptualization, validation, methodology, data analysis, writing – review and editing, writing – original draft. **Mohammed Aziz:** writing – original draft, methodology. **Mohammed Choukri:** writing – review and editing, writing – original draft. **Youssouf Ali Younous:** writing – review and editing, writing – original draft. **Amir Bouallegue:** formal analysis, supervision. **Gehan M. Elossaily:** methodology, project administration. **Abdel‐Rhman Z. Gaafar:** investigation, supervision, data curation. **Musa A. Said:** writing – review and editing, validation. **Hina Ali:** writing – review and editing, validation. **Mohamed Bnouham:** supervision, methodology, project administration.

## Funding

This work is financially supported by the Ongoing Research Funding Program, King Saud University, Riyadh, Saudi Arabia (ORF‐2026‐686).

## Disclosure

ARRIVE Guidelines: The experimentation was conducted in accordance with applicable laws, and ARRIVE guidelines.

## Ethics Statement

All procedures complied with the ethical guidelines for the care and use of laboratory animals as outlined by the US National Institutes of Health. In particular, the principles of the 3Rs—Replacement, Reduction, and Refinement—were applied to minimize animal suffering and the number of animals used.

## Consent

The authors have nothing to report.

## Conflicts of Interest

The authors declare no conflicts of interest.

## Data Availability

All data generated or analyzed during this study are included in this published article.
